# Clinical course and outcomes of type-2 diabetic patients after treatment intensification for insufficient glycaemic control - results of the 2 year prospective DiaRegis follow-up

**DOI:** 10.1186/1471-2261-14-162

**Published:** 2014-11-19

**Authors:** Peter Bramlage, Anselm K Gitt, Steffen Schneider, Evelin Deeg, Diethelm Tschöpe, the DiaRegis Study Group

**Affiliations:** Institut für Pharmakologie und präventive Medizin, Menzelstrasse 21, 15831 Mahlow, Germany; Institut für Herzinfarktforschung Ludwigshafen, Ludwigshafen, Germany; Herzzentrum Ludwigshafen, Medizinische Klinik B, Kardiologie, Ludwigshafen Germany; Stiftung “Der herzkranke Diabetiker” in der Deutschen Diabetes Stiftung, Bad Oeynhausen, Germany; Herz- und Diabeteszentrum Nordrhein-Westfalen in Bad Oeynhausen, Universitätsklinik der Ruhr Universität Bochum, Bad Oeynhausen, Germany

**Keywords:** Diabetes type 2, Glucose control, Co-morbidities, Treatment intensification, Oral antidiabetic drug, Macrovascular, Microvascular, Heart failure

## Abstract

**Background:**

In cases where antidiabetic monotherapy is unable to sufficiently control glucose levels in patients with type-2 diabetes, treatment needs to be intensified. Determining factors that may be predictors for the occurrence of comorbidities in these patients is essential for improving the efficacy of clinical diabetes care.

**Methods:**

The DiaRegis prospective cohort study included 3,810 type-2 diabetics for whom the treating physician aimed to intensify and optimise antidiabetic treatment due to insufficient glucose control. Treatment intensification was defined as increasing the dose of the originally prescribed drug, and/or selecting an alternative drug, and/or prescribing an additional drug. The aims were to monitor the co-morbidity burden of type-2 diabetic patients over a follow-up of two years, and to identify multivariable adjusted predictors for the development of comorbidity and cardiovascular events.

**Results:**

A total of 3,058 patients completed the 2 year follow-up. A substantial proportion of these patients had co-morbidities such as vascular disease, neuropathy, and heart failure at baseline. After treatment intensification, there was an increased use of DPP-4 inhibitors, insulin, and GLP-1 analogues, achieving reductions in HbA1c, fasting plasma glucose, and postprandial glucose. During the 2 year period 2.5% of patients (n = 75) died, 3.2% experienced non-fatal macrovascular events, 11.9% experienced microvascular events, and 4.3% suffered onset of heart failure. Predictors for combined macro-/microvascular complications/heart failure/death were found to be age (OR 1.36; 95% CI 1.10–1.68), prior vascular disease (1.73; 1.39–2.16), and history of heart failure (2.78; 2.10–3.68).

**Conclusions:**

Determining the factors that contribute to co-morbidities during intensive glucose-lowering treatment is essential for improving the efficacy of diabetes care. Our results indicate that age, prior vascular disease, and heart failure constitute important predictors of poor cardiovascular outcomes in patients receiving such therapy.

## Background

Monotherapy with antidiabetic drugs such as metformin is the first choice treatment strategy for type-2 diabetics for whom dietary restrictions have not managed to achieve adequate control of the condition [1–3]. Treatment escalation is left to the discretion of the consulting physician, with a variety of options available, including changes in the drug being administered or prescribing additional oral antidiabetic drugs (OADs). Injectable drugs such as GLP-1 analogues or insulin are considered to be second line alternatives. Insulin is usually recommended only when HbA1c values remain high; however, recent data have suggested that it may be safe when added earlier in the treatment process [4].

Any treatment decision should consider the patient’s particular characteristics and specific treatment goals, as well as the economic aspects [5]. This approach, however, diminishes the utility of pre-defined treatment goals and drug selection algorithms, instead favouring a treatment strategy that meets the needs of individual patients. In this regard, the European Association for the Study of Diabetes (EASD) in partnership with the American Diabetes Association (ADA) [3] and the European Society of Cardiology (ESC) [6] have provided guidelines for more patient-centred care strategies for type-2 diabetics. These guidelines are less prescriptive than prior algorithms, and owing to the lack of evidence-based inferences, their ultimate value will only become clear with comparative studies that assess real-world outcomes.

The DiaRegis prospective cohort study was designed to include patients where the treating physician aimed to intensify and optimise antidiabetic treatment due to insufficient glucose control [7]. The patients included were being treated with either one or two oral antidiabetic drugs at the time of enrolment. Treatment intensification was defined as increasing the dose of the originally prescribed drug, and/or selecting an alternative drug, and/or prescribing an additional drug. Against this background, the aim of our analysis was 1) to monitor the co-morbidity burden of type-2 diabetic patients over a follow-up of two years, and 2) to identify multivariable adjusted predictors for the development of co-morbidity and cardiovascular events.

## Methods

DiaRegis is a prospective, observational, multicentre cohort study (registry) that included 3,810 patients with type-2 diabetes under the auspices of the foundation “Der herzkranke Diabetiker” in Germany. It was conducted in accordance with Good Epidemiology Practice (GEP), and applicable regulatory requirements. The protocol was approved by the ethics committee of the Landesärztekammer Thüringen in Jena, Germany on March 4^th^ 2009, and published at baseline [7]. All patients enrolled in this registry provided written informed consent.

### Patients

Patients were enrolled in the DiaRegis registry on a consecutive basis, the mean recruitment per physician office being 13 consecutive eligible patients. At the time of enrolment, they were being treated with either one or two oral antidiabetic drugs, with the treating physician wishing to intensify treatment at the baseline visit due to inadequate glycaemic control. Such intensification included initiating an increase in the dose of the originally prescribed drug, and/or selecting an alternative drug, and/or prescribing an additional drug. Any change in treatment was left to the discretion of the treating physician without any leverage due to the study protocol.

Patients without treatment intensification or those being administered injectable antidiabetic drugs prior to baseline were not considered eligible. Further exclusion criteria included the following: patient not under regular supervision of the treating physician for the duration of the study, type-1 diabetes, pregnancy, diabetes secondary to malnutrition, infection or surgery, maturity onset diabetes of the young, known cancer or limited life expectancy, acute emergencies, participation in a separate clinical trial, and other factors preventing the patient from participating in the follow-up appointments (language skills, disabilities, hospitalisation). Patients were followed for a total of 24 months.

### Physicians

Physicians (general practitioners, internists, practitioners, and diabetologists) were selected based on a conditioned random sampling method. A physician database containing approximately 9,350 office-based physicians currently treating patients with type-2 diabetes were approached in writing. Physicians randomly distributed across all German regions with at least 150 patients with type 2 diabetes under regular medical care were invited to participate. This resulted in 313 participating physicians, representing 3.3% of the initially approached sample.

### Documentation

Patient data were entered via a secure website directly into an electronic database maintained at the Institut für Herzinfarktforschung, Ludwigshafen, Germany. This enabled online checking for plausibility and completeness. A summary of the data collected in DiaRegis, in addition to further details regarding monitoring of the collection, is provided in the design and baseline publication [7]. All data sets were included in the subsequent statistical analysis.

### Glucose control, hypoglycaemia, and co-morbidity

HbA1c, fasting glucose (overnight), and postprandial glucose (2 h after the last meal) levels were recorded at each of the follow-up visits. They were either measured in the physician’s office or recorded in specific patient diaries. No further validation or standardisation of values was attempted due to the real world-design, in addition to logistical reasons.

Data on co-morbid disease conditions and risk factors were reported by the treating physician; diagnoses were not verified independently. Crude hypoglycaemia rates were reported for history of prior hypoglycaemia (any recalled hypoglycaemia within the last 12 months) and incident hypoglycaemia (new episodes of hypoglycaemia within the 2 year follow-up). Vascular disease included coronary artery disease (CAD), stroke/transient ischaemic attack (TIA), and peripheral artery disease (PAD). Macrovascular complications included new MI, stroke, and PAD (requiring any peripheral intervention). Microvascular complications included previously unknown retinopathy, nephropathy, neuropathy, and amputation.

### Statistical analysis

The statistical analyses were performed using SAS, version 9.3 (Cary, North Carolina, USA). The distribution of continuous variables is described with medians and quartiles. Categorical parameters are presented as percentages and absolute numbers. All descriptive statistics are based on available cases. The adjusted prognostic value of patient characteristics, laboratory values at baseline, and co-morbidities on different events during the follow-up period were investigated through logistic regression analyses. The resulting odds ratios (ORs) are presented with corresponding 95% confidence intervals (CIs).

## Results

**Figure 1:**
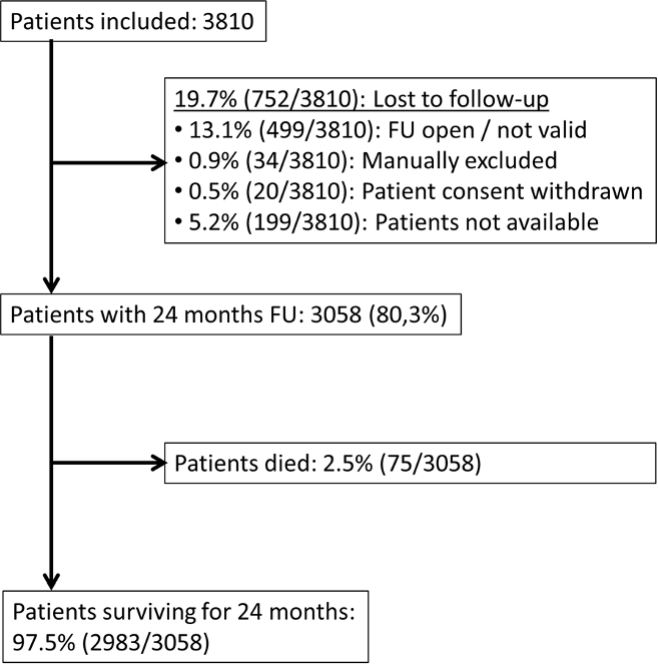
**Patient flow chart.**

A total of 3,810 patients were included in the DiaRegis registry (Figure 1). Out of the 3,058 patients that completed the 2 year follow-up period (80.3% of those enrolled), 2.5% had died (n = 75). The patients that did not complete the follow-up did not display significantly different baseline characteristics, laboratory values, co-morbidities, or pharmacotherapies to those that did complete the study (data not shown).

### Patient characteristics

Patients with a complete two-year follow-up had a median age of 66.1 (57.7–72.9) years, 46.7% were female, the median bodyweight was 88 kg (78–100), and the median duration of diabetes was 5.6 years (2.9–9.4) at baseline (Table 1). Median HbA1c was 7.4% (6.8–8.2), fasting plasma glucose was141 mg/dL (119–169) and postprandial plasma glucose was 183 mg/dL (155–220). Within the 12 months prior to inclusion into DiaRegis, 1.4% of patients had experienced an episode of severe hypoglycaemia. A substantial proportion of patients (24.3%) had known vascular disease with CAD and/or prior stroke or TIA and/or PAD). Furthermore, 14.5% had known autonomic or peripheral neuropathy and 9.9% had prior heart failure (HF).

**Table 1:** **Patient characteristics at baseline**

	Patients included (n = 3810)	Patients with a 2 year FU* (n = 3058)
Age (years)	65.9 (57.6–72.9)	66.1 (57.7–72.9)
Female gender (%)	46.7	46.7
Body weight (kg)	88 (78–100)	88 (78–100)
Diabetes duration (years)	5.5 (2.9–9.4)	5.6 (2.9–9.4)
Lipid values		
LDL-C (mg/dL)	120 (98–145)	119 (96–145)
HDL-C (mg/dL)	47 (40–57)	47 (40–56)
TG (mg/dL)	176 (127–242)	175 (127–241)
TC (mg/dL)	204 (175-232)	211 (181-235)
Blood pressure (mmHg)	137/80	137/80
Blood glucose		
HbA1c (%)	7.4 (6.8–8.3)	7.4 (6.8–8.2)
FPG (mg/dL)	142 (119–171)	141 (119–169)
PPG (mg/dL)	185 (155–221)	183 (155–220)
Hypoglycaemia requiring assistance (%)	1.2	1.4
Concomitant disease (%)		
Prior MI (%)	6.0	5.8
Prior stroke/TIA (%)	4.6	4.8
HF (%)	9.9	9.9
PAD (%)	6.0	6.2
Prior amputation (%)	0.9	0.9
Any neuropathy (%)	15.9	14.5
Any retinopathy (%)	4.2	4.2
Vascular disease (%)**	24.0	24.3
Cardiovascular pharmacotherapy		
ACEi	50.0	50.5
ARB	21.8	21.9
Betablocker	46.6	46.5
CCB	24.9	25.4
Diuretic	41.2	42.0
ASA	33.6	33.8
Statin	42.2	42.6
Antidiabetic therapy post baseline		
Metformin (%)	84.5	84.5
Sulfonylureas (%)	26.2	27.2
Glucosidase inhibitors (%)	2.3	2.6
Glinides (%)	6.0	5.3
DPP-4 inhibitors (%)	38.8	39.3
Glitazones (%)	10.3	10.1
GLP-1 analogues (%)	9.2	9.7
Insulin (%)	17.3	17.6

*Legend:* *Including those that died during follow-up; **vascular disease includes CAD, prior stroke/TIA, and/or PAD; FU, follow-up; TG, triglycerides; FPG, fasting plasma glucose; PPG, postprandial plasma glucose; MI, myocardial infarction; TIA, transitory ischemic attack; HF, heart failure; PAD, peripheral artery disease; ACEi, angiotensin-converting enzyme inhibitor; ARB, angiotensin receptor blocker; CCB, calcium channel blocker; ASA, acetylsalicylic acid; DPP, dipeptidylpeptidase; GLP, glucagon-like peptide; SU, sulfonylurea.

### Pharmacotherapy and the course of glucose control

**Figure 2:**
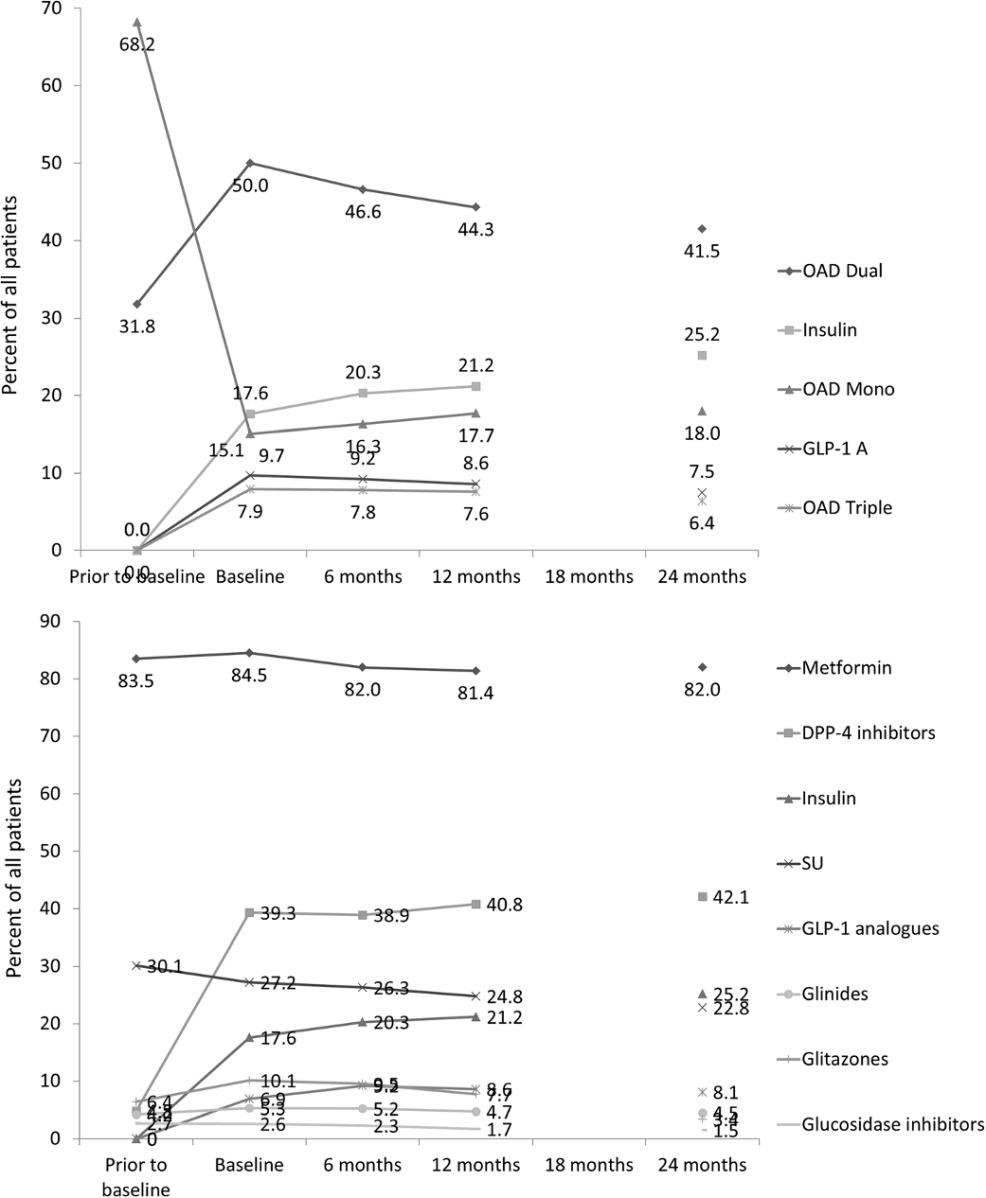
**Change in therapy during follow-up period.** Upper graph: Combinations of OADs, insulin, and GLP-1 analogues; Lower graph: differences in treatment therapies.

As per the protocol, patients were receiving either mono- or dual combination oral antidiabetic treatment at the time of enrolment. There was a predominance of oral monotherapy (68.2%) at this point, with many of these patients being switched to combination therapies (dual OAD 50.0%; triple OAD 7.9%) at the baseline visit (Figure 2, upper graph). This was accompanied by a significant increase in prescription of DPP-4 inhibitors (4.8% v 39.3%), insulin (0% v 17.6%), and GLP-1 analogues (0% v 6.90%) (Figure 2, lower graph). After the initial adaptation of therapy at baseline, additional changes in pharmacotherapy were moderate, with a gradual further increase in insulin use (up to 25.2% at 2 years) and a decline in dual oral combinations (50.0% after baseline visit down to 41.5% at 2 years).

**Figure 3:**
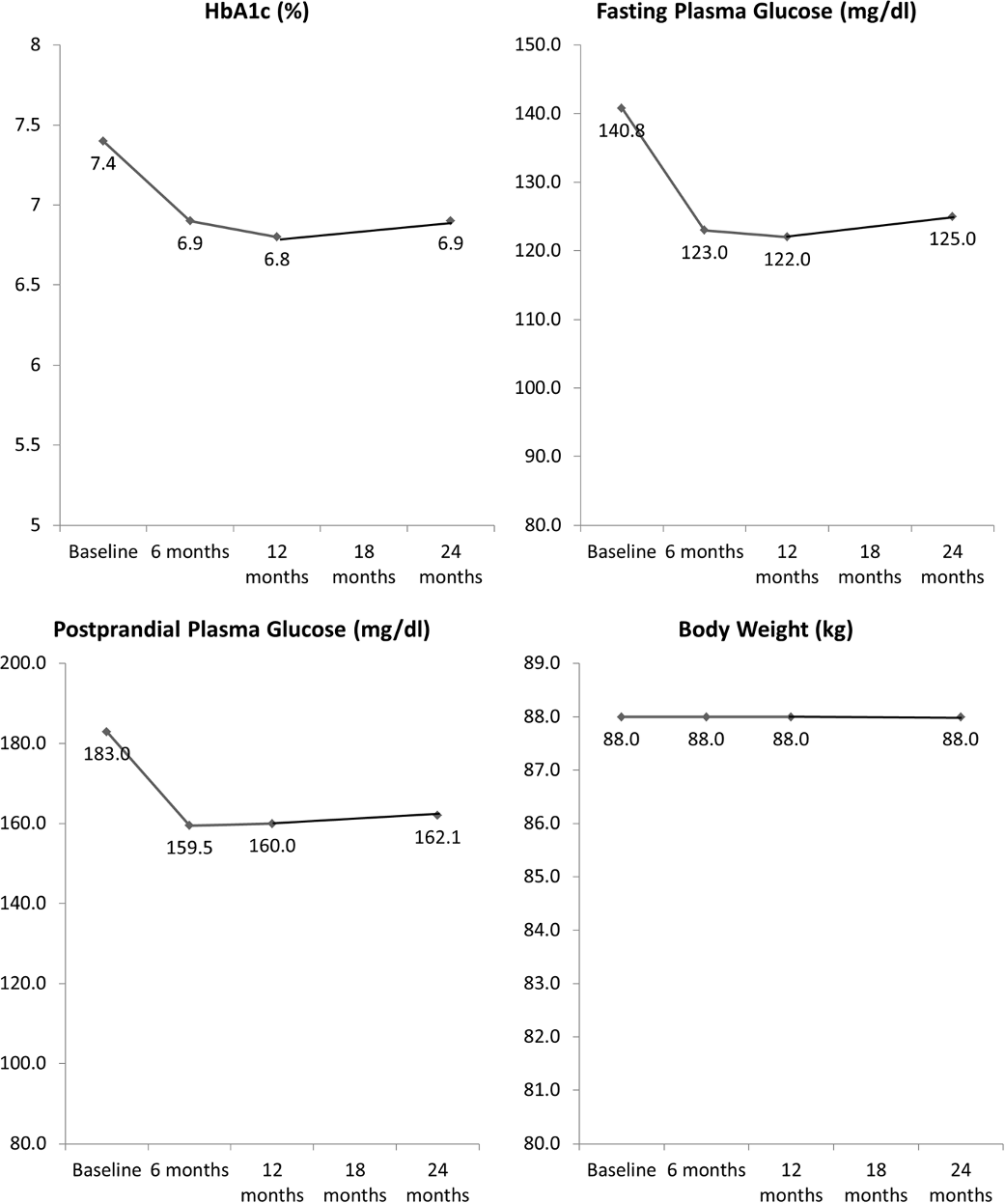
**Course of median glucose values and bodyweight during follow-up.** Legend: changes in Hb1Ac, fasting plasma glucose, postpradial glucose, and body weight over the 2 year follow-up (n = 3058).

The treatment switch at baseline resulted in a considerable reduction in median HbA1c levels (7.4% to 6.9%), fasting plasma glucose (140.8 mg/dL to 123.0 mg/dL), and postprandial glucose (183.0 mg/dL to 159.5 mg/dL) in the first 6 months of the follow-up period (Figure 3). These values were found to be relatively stable through to the final follow-up visit at 2 years. The median body weight (88 kg) in the overall population was stable throughout the 2 year period.

### Adverse event burden of type-2 diabetic patients over the two year follow-up

During the 2 year follow-up period, 75 patients (2.5%) died. A further 3.2% experienced a macrovascular event, defined as CAD, stroke/TIA, or PAD (Table 2). Microvascular complications were more frequent (11.9%). Incident HF was reported in 4.3% of the patients during the 2-year follow up.

**Table 2:** **Events and newly diagnosed co-morbidities during follow-up**

	Patients with a 2 year FU*	
	Total patients (n)	% of those with FU
Death	75/3058	2.5
Macrovascular complications	94/2979	3.2
CAD	24/2979	0.8
Stroke/TIA	42/2979	1.4
PAD	37/2979	1.2
Microvascular complications	355/2979	11.9
Any retinopathy	98/2979	3.3
Any nephropathy	3/2979	0.1
Any neuropathy	305/2979	10.2
Amputation	9/2979	0.3
HF	128/2979	4.3
Non-fatal events combined	490/2979	18.5
All events	565/3058	18.5

*Legend:* *Including those that died during follow-up; CAD, coronary artery disease; TIA, transitory ischaemic attack; PAD, peripheral artery disease; HF, heart failure; FU, follow-up.

### Multivariable adjusted predictors for the development of comorbidity and vascular events

We identified multivariable adjusted predictors for macro- and microvascular events, as well as HF (Table 3). There was a consistent trend towards increased event rates in patients with prior vascular disease, HF, and longer diabetes duration. Predictors for the combined endpoint (macro-/microvascular complication/HF/death) were age (OR 1.36; 95% CI 1.10–1.68), prior vascular disease (1.73; 1.39–2.16), and history of prior HF (2.78; 2.10–3.68).

**Table 3:** **Multivariable adjusted predictors of events (n = 3058)**

	MAC/MIC/HF/Death OR (95% CI)	MAC* OR (95% CI)	MIC* OR (95% CI)	HF OR (95% CI)
Patient characteristics at baseline				
Age ≥ median	**1.36 (1.10–1.68)**	1.15 (0.71–1.86)	1.15 (0.89–1.47)	**2.25 (1.21–4.16)**
Male vs. female	1.17 (0.96–1.43)	1.36 (0.87–2.14)	1.21 (0.96–1.54)	0.74 (0.45–1.20)
BMI ≥ median	1.01 (0.83–1.23)	0.69 (0.45–1.07)	1.11 (0.87–1.41)	0.85 (0.52–1.38)
Diabetes duration ≥ median	**1.29 (1.06–1.57)**	**1.94 (1.24–3.05)**	**1.33 (1.05–1.68)**	0.91 (0.56–1.48)
Laboratory values				
HbA1c ≥ median	1.12 (0.90–1.39)	0.92 (0.57–1.46)	**1.29 (1.00–1.66)**	1.14 (0.66–1.96)
FPG ≥ median	1.09 (0.89–1.35)	1.00 (0.63–1.59)	1.18 (0.92–.51)	0.60 (0.34–1.04)
Complications				
Vascular disease*	**1.73 (1.39–2.16)**	**2.72 (1.72–4.31)**	**1.58 (1.22–2.06)**	1.14 (0.66–1.96)
HF	**2.78 (2.10–3.68)**	**1.97 (1.13–3.43)**	**1.53 (1.07–2.18)**	**--**
Severe hypoglycaemia	1.24 (0.59–2.62)	0.44 (0.06–3.38)	1.88 (0.85–4.14)	1.27 (0.35–4.60)

*Legend:* MAC, macrovascular complication; MIC, microvascular complication; HF, heart failure; BMI, body mass index; FPG, fasting plasma glucose. *Vascular disease includes CAD, stroke/TIA, and PAD; MICs include previously unknown retinopathy, nephropathy, neuropathy, and amputation; MACs include MI, stroke/TIA, and PAD (any peripheral intervention). Bold ORs (95%CI) reflect significant predictors of events.

Particularly noteworthy were the increased risk for macrovascular events in patients with a diabetes duration of at least 5.6 years (median; OR 1.94; 95% CI 1.24–3.05) and the increased risk of HF in the elderly (OR 2.25; 95% CI 1.21–4.16).

## Discussion

In the present study, we monitored type-2 diabetes patients for 2 years after intensification of their antidiabetic treatment, in order to identify comorbidities and risk factors that might be associated with poor outcome. At baseline, glycaemic control after being treated with either one or two OADs was considered to be insufficient. Therefore, upon enrolment into this study, patients were commonly switched to combination therapies, which often included the use of DPP-4 inhibitors, insulin, and GLP-1 analogues. We observed that these intensified regimens were typically accompanied by a significant reduction in median HbA1c levels. However, patients continued to exhibit considerable risk for death (2.5%), macrovascular complications (3.2%), microvascular complications (11.9%), and HF (4.3%).

Although cardiovascular co-morbidities associated with diabetes treatment were identified, it should be noted that a substantial proportion of patients had known vascular disease (24.3%) or HF (9.9%; any severity) at baseline. Nevertheless, we observed a considerable burden of incident comorbidities, which is in good agreement with previous observations. While there are many studies demonstrating a link between diabetes and increased cardiovascular risk, there is also evidence of an association with death from other causes. A study by Gordon-Dseagu et al. found that mortality due to respiratory disease or some cancers was greater in diabetic patients [8]. Furthermore, a recent analysis of 820,900 patients included in prospective studies demonstrated increased overall mortality in patients with diabetes, in addition to increases in death from a variety of cancers, vascular causes, renal disease, and other factors [9]. There is the possibility that differences in these risks originate not only from dysglycaemia, but also from the particular treatment strategy selected. For example, a number of antidiabetic drugs, including insulin, have been linked to weight gain [10], and there is an ongoing debate regarding the safety of certain drugs such as sulfonylureas [11], resulting in an FDA request for macrovascular endpoint studies. Two such studies, the EXAMINE and SAVOR TIMI-53 trials [12, 13], demonstrated no increased risk with gliptin use. This was in agreement with a meta-analysis by Gooßen et al., who reported few associations between risk and gliptin monotherapy; the data regarding combination therapies were less clear however [14]. Therefore, it appears that further in depth study is required in order to delineate which patient characteristics or drug types contribute to the observed variability in cardiovascular risk during intensified treatment strategies.

With regard to risk factors that might predict a poor cardiovascular outcome during intensified treatment, we observed a consistent trend of increased macrovascular and microvascular events in patients with prior vascular disease, longer diabetes duration, and HF at baseline. Moreover, we found that predictors for a combined cardiovascular endpoint (i.e., macro-/microvascular complications, HF, and death) included age, prior vascular disease, and HF. Notably, these risk factors were also reported in the ACCORD trial, which demonstrated that patients with previous cardiovascular events or multiple cardiovascular risk factors had a higher mortality rate on undergoing intensive glucose control therapy in comparison to standard treatment [15]. The VA diabetes trial also identified prior cardiovascular events to be a predictor for primary events during intensive antidiabetic therapy [16]. The ADVANCE randomised controlled trial compared the use of gliclazide-centred intensive therapy with standard treatment for reducing HbA1c levels in type-2 diabetics with a history of vascular complications [17]. In contrast with the ACCORD study, little difference was found in the rate of death from cardiovascular causes. However, it should be noted that the specific intensive therapies administered in these two trials differed, with a higher proportion of patients in the ACCORD trial being treated with insulin. It has been suggested that this may be a factor in the increased mortality found during this latter study [18]. The ADVANCE trial also noted that nephropathy was demonstrated to be less prevalent in patients undergoing intensive therapy [17]. This is particularly interesting owing to the increased cardiovascular risk that has been demonstrated for patients displaying indications of renal disease [19, 20]. However, in the present study, microvascular complications, in particular neuropathy and retinopathy, were found to occur more frequently in particular sub-groups of patients receiving intensive therapy, with longer diabetes duration, prior vascular disease, and HF all being predictors of such events (Table 3). In the UK Prospective Diabetes Study (UKPDS) completed in 1998, overall microvascular complications were reported to be less prevalent in patients receiving intensive antidiabetic therapies based on sulfonylurea-insulin, a risk which was sustained through the 10 year follow-up period [21, 22]. Incidence of macrovascular events, on the other hand, was not shown to be significantly different between the two groups in the original trial period. However, at long term follow-up, there was some evidence of a lower risk in patients who had received intensive treatment.

In the present study, we demonstrated an increase in macrovascular events for those individuals with diabetes duration of greater than 5.6 years at baseline. Data obtained during the VA diabetes trial led to the hypothesis that intensive therapy might reduce cardiovascular events if initiated during the first 15 years after a diabetes diagnosis. After this point, however, the results indicated that the risk of cardiovascular complications increases [16].

This study had some limitations. The list of predictors for cardiovascular complications investigated is by no means comprehensive. By evaluating patient characteristics in more detail, it may be possible to identify further factors of interest. In addition, by design, the patients included in this study required adjustments in their treatment regimens in order to achieve glycaemic control. Thus, additional changes in treatment with the aim of reaching acceptable HbAc1 levels during the 2 year follow-up period may have impacted the results. Finally physicians participating in this registry where chosen at random from a large database of which some declined. So there is a potential selection bias towards those more enthusiastic about treatment intensification.

## Conclusions

In cases where antidiabetic monotherapy is unable to sufficiently control glucose levels in patients with type-2 diabetes, treatment needs to be intensified. Determining factors that may be predictors for the occurrence of cardiovascular complications in these patients is essential for improving the efficacy of clinical diabetes care. In the present study we have shown that age, prior vascular disease, and HF may be predictors of a poor outcome in patients receiving intense antidiabetic treatment. Further investigation into these factors, in addition to identification of others, should allow for treatment regimen to be based on individual patients’ characteristics, potentially decreasing the incidence of adverse events.

## Abbreviations

ACEi: Angiotensin-converting enzyme inhibitor
ACCORD: Action to control cardiovascular risk in diabetes
ADVANCE: Action in diabetes and vascular disease: preterax and diamicron MR controlled evaluation
ARB: Angiotensin receptor blocker
ASA: Acetylsalicylic acid
CAD: Coronary artery disease
CCB: Calcium channel blocker
DPP-4: Dipeptidylpeptidase-4
EXAMINE: Examination of cardiovascular outcomes with alogliptin versus standard of care
FPG: Fasting plasma glucose
FU: Follow-up
GEP: Good epidemiology practice
GLP: Glucagon-like peptide
HF: Heart failure
MAC: Macrovascular complication
MI: Myocardial infarction
MIC: Microvascular complication
OAD: Oral antidiabetic drug
PAD: Peripheral artery disease
SAVOR TIMI-53: Saxagliptin assessment of vascular outcomes recorded in patients with diabetes mellitus, thrombolysis in myocardial infarction 53
TIA: Transient ischaemic attack
UKPDS: UK prospective diabetes study
VA: Veteran’s association.
